# Multi-omics analysis reveals that alginate oligosaccharides mitigate ochratoxin A-induced renal impairment in mice and is relevant to the regulation of PPAR signaling

**DOI:** 10.3389/fvets.2025.1702799

**Published:** 2026-01-12

**Authors:** Xueqing Ye, Yue Zhao, Qinghua Yao, Xu Zhang, Sibing Li, Wenchao Liu

**Affiliations:** Department of Animal Science, College of Coastal Agricultural Sciences, Guangdong Ocean University, Zhanjiang, China

**Keywords:** alginate oligosaccharides, multi-omics, nephrotoxicity, ochratoxin A, PPAR signaling

## Abstract

**Introduction:**

Ochratoxin A (OTA) is a core environmental toxin that induces kidney injury by interfering with glomerular filtration, antioxidant defense, and tubular transport functions. Alginate oligosaccharides (AOS), as active substances from marine, carry natural antioxidant, anti-inflammatory and other biological activities. The purpose of this study is to explore the molecular network of AOS against nephrotoxicity caused by OTA.

**Methods:**

A total of 36 5-week male mice were randomly divided into three groups: the CON group, the OTA group (250 μg/kg B.W. OTA) and the AOS + OTA group (400 mg/kg B.W. AOS +250 μg/kg B.W. OTA). The treatment was continued for 21 d.

**Results:**

OTA induced renal injury in mice, manifested by glomerular capsule blurring, lymphocytic infiltration, and mitochondrial damage in tubular epithelial cells. Treatment with AOS significantly alleviated these pathological changes. Multi‑omics analysis revealed that AOS activated the PPAR signaling pathway, upregulating key genes (Aldehyde Dehydrogenase 1 Family Member A3 (*Aldh1a3*), Carbamoyl-phosphate synthase 1 (*Cps1*), Cytochrome c oxidase subunit 8B (*Cox8b)*), which drove the accumulation of protective metabolites such as L‑arginine and carnosine. This protective process involved coordinated regulation of amino acid metabolism, mTOR signaling, and PPAR pathways, illustrating a novel metabolic‑transcriptional network through which AOS mitigates OTA‑induced nephrotoxicity.

**Discussion:**

This study reveal that AOS antagonizes OTA-induced nephrotoxicity in mice through PPAR signaling axis, thus providing new insight into the renal protection mechanism of marine active substances.

## 1 Introduction

Ochratoxin is a secondary metabolite produced by strains of Aspergillus and Penicillin, widely present in various feed raw materials, and has multiple toxicological effects on the organism (1). Ochratoxin A (OTA) is one of the most widely distributed, toxic and harmful toxins among the different kinds of ochratoxin, and its toxicity is second only to aflatoxin B_1_ (2). OTA exposure causes toxicity in a variety of organs, but it is important to note that the kidney is the primary target organ for OTA attacks (3). The kidney, as an important organ, can eliminate metabolites and poisons by producing urine, and at the same time, it can reabsorb other useful substances to adjust the electrolyte balance and acid–base balance in the body, thus ensuring the steady state of the internal environment of the body and making the metabolism run normally (4). However, it is worth noting that OTA is extremely toxic to the kidney, which is not conducive to OTA metabolism by affecting the structure, filtration function and reabsorption function of the kidney. Research indicates that OTA interferes with kidney function by reducing the glomerular filtration rate (5). Moreover, it has been found that OTA diminishes the antioxidant capacity of body, which reduces the clearance of reactive oxygen species (ROS) (6). OTA can also lead to renal dysfunction by destroying the transport of organic ions on the brush edge of proximal tubule epithelial cells (5). Besides, OTA accumulates in proximal tubule epithelial cells, causing adverse effects such as oxidative damage, apoptosis and inflammatory reaction (7).

As a natural active macromolecule from the ocean, seaweed polysaccharide has been proved to hold a wide range of biological activities and therapeutic functions (8). Alginate oligosaccharides (AOS), as a kind of oligosaccharide fragment obtained by enzymolysis or acidolysis of seaweed polysaccharides, has the advantages of strong water solubility, high bioavailability and low toxic and side effects compared with traditional marine polysaccharides (9). In recent years, it has been found that AOS can promote the synthesis of glutathione (GSH), and alleviate the oxidative stress of cells by activating the nuclear factor E2-related factor 2 (Nrf2)/heme oxygenase-1 (HO-1) pathway (10). AOS reduces the levels of proinflammatory factors such as tumor necrosis factor-α (TNF-α) and interleukin-6 (IL-6) via inhibiting the inflammatory cascade mediated by toll-like receptor 4 (TLR4) (11, 12). The existing research described that AOS can weaken the pathological phenomena of renal tubular sclerosis, atrophy, edema and vacuolar degeneration. At the same time, AOS can also improve renal interstitial fibrosis with inflammatory cell infiltration (13). Studies have shown that fucoidan from the same seaweed as AOS can reduce the levels of serum creatinine and urea nitrogen in mice, increase the clearance rate of creatinine, and help to improve renal function and maintain renal excretion (14). In addition, seaweed-derived saccharides have been reported to inhibit the excessive mitochondrial autophagy in kidney, improve intestinal barrier function, and then prevent secondary renal injury (15). Although the biological activity of AOS has been widely verified, the research on its therapeutic effect on kidney diseases is still in its infancy, and the specific molecular mechanisms such as mitochondrial ultrastructure changes and metabolite accumulation in kidney induced by OTA have not been clarified.

Currently, the potential mechanism of OTA nephrotoxicity involves the interaction of many factors, so it is difficult to distinguish the core driving factors from other accompanying phenomena in a single omics data, and it is also impossible to analyze the multi-layer regulatory network that dominates the complex phenotype. It is worth noting that one of the insights of the multi-omics data set is the molecular response across multiple organ systems (16). Metabolomics can provide abundant information about the biochemical state of organs (17, 18), and transcriptomics can predict the changes of gene expression, thus revealing the relevant molecular mechanisms (19). In addition, metabolomics can reflect and amplify the changes of transcriptomics, and metabolites regulate gene transcription (20). The combination of transcriptomics and metabolomics is able to better understand the mechanism of cell physiological activities at molecular and biochemical levels (21). However, at present, AOS is generally found to have anti-inflammatory and antioxidant effects through phenotypic studies *in vitro* and *in vivo*, while the renal protective mechanism of AOS based on multi-omics analysis is lacking. Therefore, we assayed the morphological damage of kidney by H&E staining, located the changes of subcellular structure by transmission electron microscope (TEM), determined the accumulation or decrease of related metabolites using metabolomic analysis, and understanded the different expression of key genes related to kidney function using transcriptomics, in order to elucidate the protective role and mechanism of action of AOS on OTA-induced nephrotoxicity.

## 2 Materials and methods

### 2.1 Animal and experimental design

The animal study protocol was approved by Coastal Agricultural Sciences of Guangdong Ocean University (Approval No. 20211009, Zhanjiang, Guangdong, China). Five-week-old C57BL/6J male mice were purchased from Beijing HFK Bio-Technology. Co., Ltd. (Beijing, China). All mice were under controlled environmental conditions with a 12-h light–dark cycle; 45–60% relative humidity and temperature of 25 ± 2 °C. After a week acclimatization period, the mice were divided into three groups, each with 12 mice: (1) the control group (CON), mice receiving gavage with 1 mL/kg body weight (B.W.) distilled water and 1 mL/kg B.W. 0.1 M sodium bicarbonate; (2) the OTA group, mice receiving gavage with OTA at 250 μg/kg B.W. containing 1 mL/kg B.W. 0.1 M sodium bicarbonate; (3) the AOS + OTA group, mice given AOS at 400 mg/kg B.W. 1 mL/kg B.W. distilled water and OTA at 250 μg/kg B.W. containing 1 mL/kg B.W. 0.1 M sodium bicarbonate. The AOS and OTA treatment was continued for 21 days, and the OTA and AOS additive levels refer to the previous studies (22, 23). At the end of the experiment, animals were sacrificed by cervical dislocation. Kidney samples of 6 mice in each group were randomly selected for H&E staining and TEM, and kidney samples of three mice were taken out for transcriptomics and metabolomics analysis. The AOS was obtained from Qingdao BZ OLIGO Biotech Co., Ltd. (Qingdao, China), its purity is 90% and the molecular weight is 2.83 kDa. Monosaccharide composition analysis using high-performance liquid chromatography (HPLC) revealed that AOS consists of glucosamine, galacturonic acid, galactosamine, glucose, and galactose, with molar percentage ratios of 19.29:4.95:13.91:5.11:56.73. The OTA with a purity greater than or equal to 98% was purchased from Pribolab (Qingdao, China).

### 2.2 Histopathological assessment

H&E staining was performed as previously described (24). Fresh kidney tissue samples were fixed in 4% paraformaldehyde solution for 48 h, and then were embedded in paraffin: dehydrated by gradient ethanol (70, 80, 90, 95, 100%) in turn, and transparent with xylene, and then soaked in liquid paraffin and embedded into tissue wax blocks. The wax block was continuously cut into 4 μm thick slices with a slicer and mounted on a glass slide. These slices were stained with hematoxylin and eosin (H&E) by Wuhan Sevier Biotechnology Co., Ltd. (Wuhan, China).

### 2.3 Transmission electron microscope

In order to evaluate the ultrastructural pathological changes of renal tissue, we carried out transmission electron microscope analysis. As mentioned above (25), the fresh kidney tissues were fixed with 2.5% glutaraldehyde solution. The fixed tissue was rinsed with 0.1 M PBS, and then fixed with 1% osmium tetroxide (also prepared with phosphate buffer) at 4 °C for 2 h. Subsequently, the tissue was dehydrated by gradient ethanol, embedded, polymerized, dyed and sealing. The images were observed and recorded under TEM and analyzed by Image J software (v1.53, MD, United States).

### 2.4 Metabolomics analysis

Accurately weigh 100 μg of kidney tissue into a 2 mL centrifuge tube, add 1 mL tissue extract (75% 9:1 methanol: chloroform, 25% water) and the same amount of steel balls, grind them in a tissue grinder for 120 s, then perform ultrasonic treatment at room temperature for 30 min, centrifuge at 12,000 rpm for 10 min at 4 °C, take the supernatant, concentrate and dry it. Then, add 200 μL 50% acetonitrile solution prepared with 2- amino-3-(2-chlorophenyl) propionic acid (4 ppm) was redissolve the sample, and the supernatant was filtered by 0.22 μm membrane, and the filtrate was transferred to a detection bottle for LC-MS detection. The raw data were firstly converted to mzXML format by MSConvert in Proteowizard software package (v3.0.8789) and processed using XCMS for feature detection, retention time correction and alignment. *p*-value was calculated according to statistical test, variable importance in the projection (VIP) was calculated by orthogonal partial least squares discriminant analysis (OPLS-DA) dimension reduction method, and fold change was used to calculate the component difference multiple, so as to measure the influence of each metabolite component content on sample classification and discrimination. Differential metabolites were identified using thresholds of VIP >1, |log2 fold change| ≥1, and adjusted *p*-value <0.05.

### 2.5 Transcriptomic sequencing and analysis

Suzhou Panomix Biomedical Tech Co., Ltd. (Suzhou, China) used the second-generation sequencing technology to sequence the samples after RNA extraction, purification, database building and other steps, based on Illumina sequencing platform. The filtered Reads were compared with the reference genome by using the upgraded version of Hisat2^1^ software. Subsequently, the differentially expressed genes (DEGs) were screened, that is, the expression difference multiple |log2 fold change| >1, with a significant *p*-value <0.05. Blast2go was used for Gene Ontology (GO) enrichment analysis, and Kyoto Encyclopedia of Genes and Genomes (KEGG) analysis were performed using KAAS.

### 2.6 Statistical analysis

All results are presented as mean ± standard error of the mean (SEM). Statistical analyses were performed using GraphPad Prism software version 9.5.0. *p* < 0.05 was considered statistically significant, and 0.05 < *p* < 0.1 with considered trend towards significance.

## 3 Results

### 3.1 Effect of AOS on renal histomorphology in mice exposed to OTA

**Figure 1:**
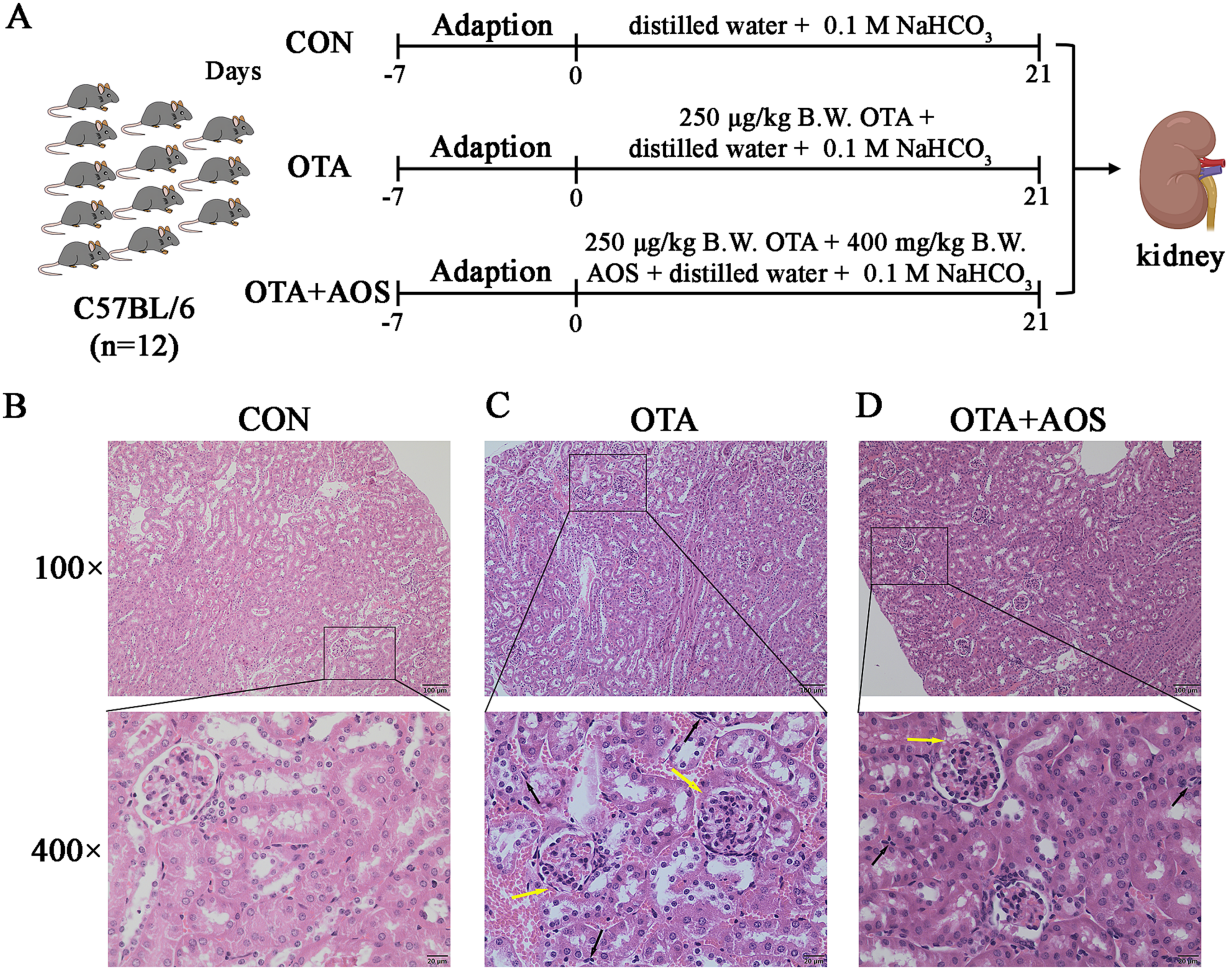
Effect of alginate oligosaccharides (AOS) on renal histomorphology in mice exposed to ochratoxin A (OTA). **(A)** Overview of experimental grouping of OTA and AOS on mouse kidney. **(B–D)** H&E staining images of kidney sections in indicated CON, OTA and OTA + AOS groups mice. Black arrow represents inflammatory cell infiltration. Yellow arrow represents the blurring of balloon lumen. The scar bar is 100 μm of 100× and 25 μm of 400×. CON, 1 mL/kg B.W. H_2_O + 1 mL/kg B.W. 0.1 M sodium bicarbonate; OTA, 1 mL/kg B.W. H_2_O + 1 mL/kg B.W. 0.1 M sodium bicarbonate + 250 μg/kg B.W. OTA; OTA + AOS, 1 mL/kg B.W. H_2_O + 1 mL/kg B.W. 0.1 M sodium bicarbonate + 250 μg/kg B.W. OTA + 400 mg/kg B.W. AOS.

The schematic diagram of the mouse grouping and treatment process is shown in Figure 1A. H&E staining was performed on three groups to investigate the effects of AOS on OTA-induced mouse kidneys. In the control group, the kidney structure was clear and the glomerular morphology was regular. The epithelial cells of proximal convoluted tubule and distal convoluted tubule are arranged closely, and there is no inflammatory cell infiltration (Figure 1B). OTA led to the blurring of balloon lumen, the infiltration of renal cortical lymphocytes, and accompanied by slight edema of renal tubular epithelial cells, which was alleviated by AOS treatment (Figure 1C). After AOS intervention, the degree of renal tissue damage in mice was reduced. The glomerular structure basically recovered clearly, the phenomenon of blurred balloon lumen was alleviated, and the infiltration range and density of inflammatory cells were significantly reduced (Figure 1D).

### 3.2 Effect of AOS on ultrastructure of renal mitochondria in mice exposed to OTA

**Figure 2:**
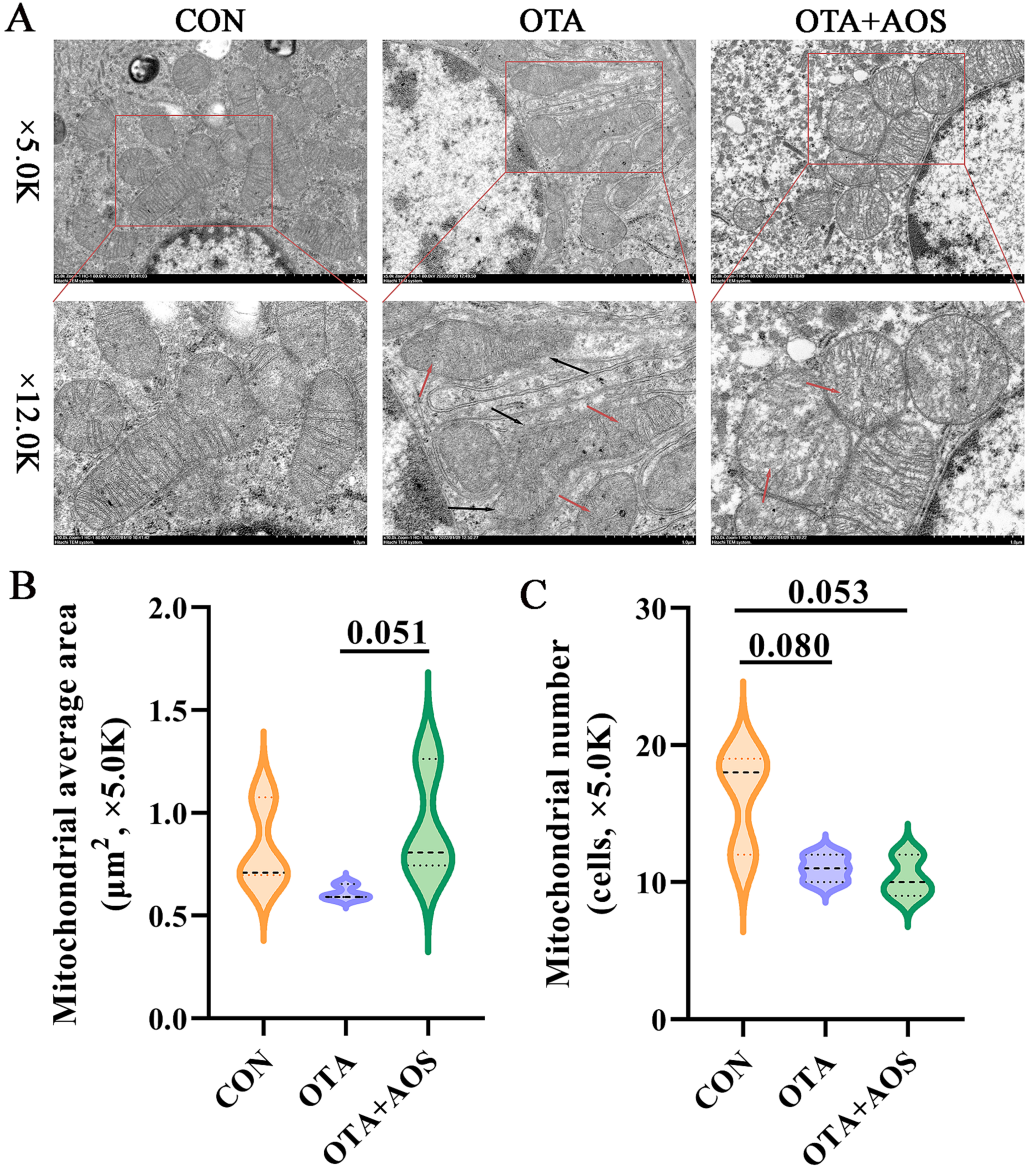
Effect of alginate oligosaccharides (AOS) on ultrastructure of kidney mitochondria in mice exposed to ochratoxin A (OTA). **(A)** Transmission electron microscope (TEM) images of mice’s kidney mitochondria. The scar bar is 5.0 K and 12.0 K. Red arrow represents the disorder of mitochondrial cristae. Black arrow represents the blurring or disappearance of the mitochondrial bilayer membrane structure. **(B,C)** Average area and number of mitochondria at 5.0 K. CON, 1 mL/kg B.W. H_2_O + 1 mL/kg B.W. 0.1 M sodium bicarbonate; OTA, 1 mL/kg B.W. H_2_O + 1 mL/kg B.W. 0.1 M sodium bicarbonate + 250 μg/kg B.W. OTA; OTA + AOS, 1 mL/kg B.W. H_2_O + 1 mL/kg B.W. 0.1 M sodium bicarbonate + 250 μg/kg B.W. OTA + 400 mg/kg B.W. AOS. Data are expressed as mean ± SEM.

The results of transmission electron microscope analysis of mitochondrial ultrastructure (Figure 2A) clearly reveal the effects of different treatment groups on renal tubular mitochondria. In the control group, the mitochondrial structure is normal, and the cristae structure is clear and numerous under normal circumstances. OTA treatment led to significant mitochondrial damage, mainly manifested as: the double membrane of mitochondria was blurred, and the internal ridge structure of a large number of mitochondria was blurred or even completely disappeared. Compared with OTA group, after AOS treatment, the mitochondrial damage was obviously repaired, the mitochondrial swelling was reduced, and the contour returned to a more normal shape. Quantitative analysis of mitochondrial morphological structure shows that the number of mitochondria in OTA group tends to decrease compared with the control group (*p* = 0.080). Compared with the OTA group, the mitochondrial area of AOS group tends to increase under 5.0 K visual field (*p* = 0.051) (Figures 2B,C).

### 3.3 Effect of AOS on renal metabolomics in mice exposed to OTA

**Figure 3:**
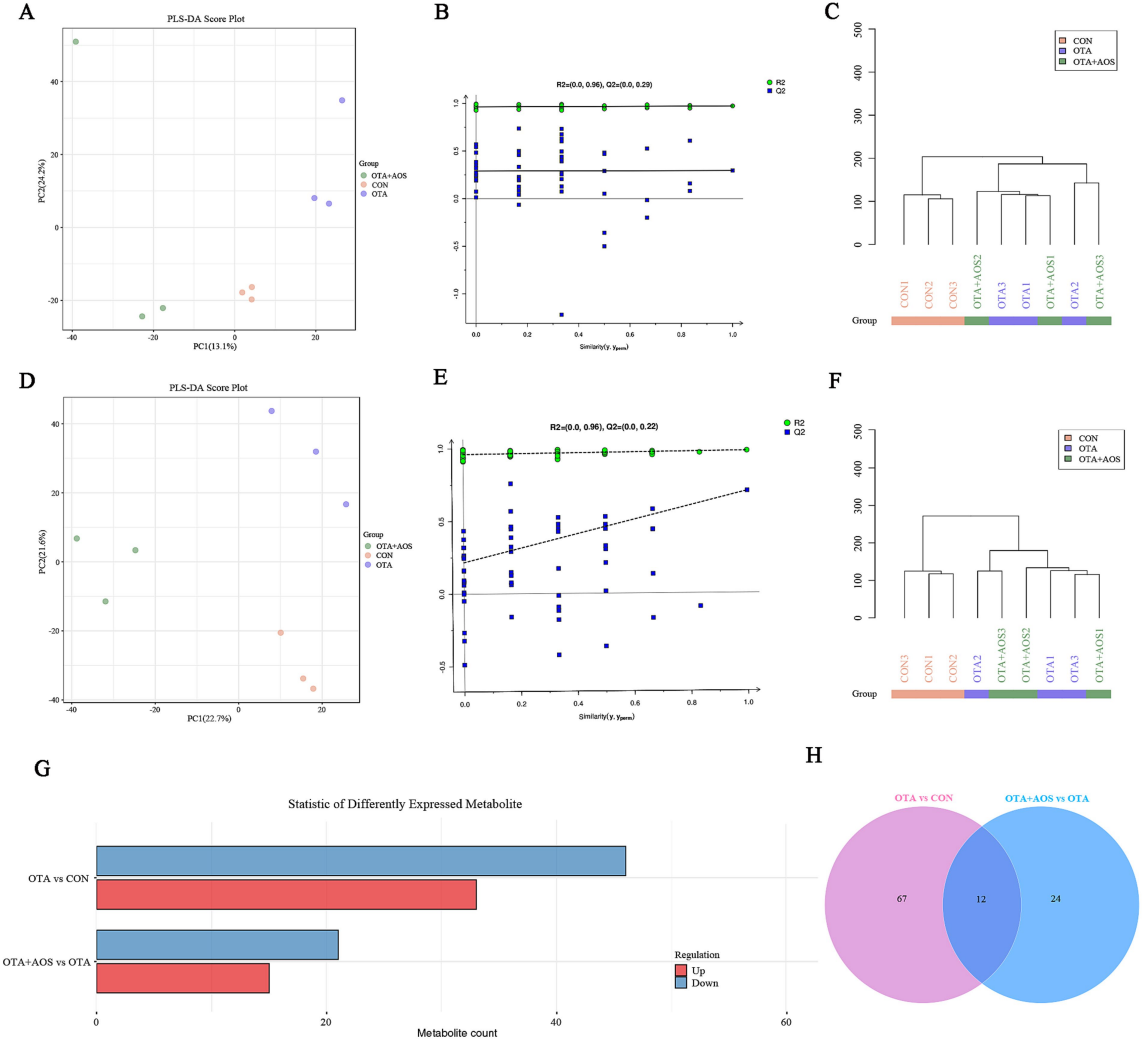
Effect of alginate oligosaccharides (AOS) on kidney metabolomics in mice exposed to ochratoxin A (OTA). **(A,B)** Partial least squares discriminant analysis (PLS-DA) and model verification in positive ion mode. **(C)** Sample hierarchical clustering tree in positive ion mode. **(D,E)** Partial least squares discriminant analysis (PLS-DA) and model verification in negative ion mode. **(F)** Sample hierarchical clustering tree in negative ion mode. **(G)** Bar chart of differential metabolites number. **(H)** Venn diagram of differential metabolites. CON, 1 mL/kg B.W. H_2_O + 1 mL/kg B.W. 0.1 M sodium bicarbonate; OTA, 1 mL/kg B.W. H_2_O + 1 mL/kg B.W. 0.1 M sodium bicarbonate + 250 μg/kg B.W. OTA; OTA + AOS, 1 mL/kg B.W. H_2_O + 1 mL/kg B.W. 0.1 M sodium bicarbonate + 250 μg/kg B.W. OTA + 400 mg/kg B.W. AOS.

**Figure 4:**
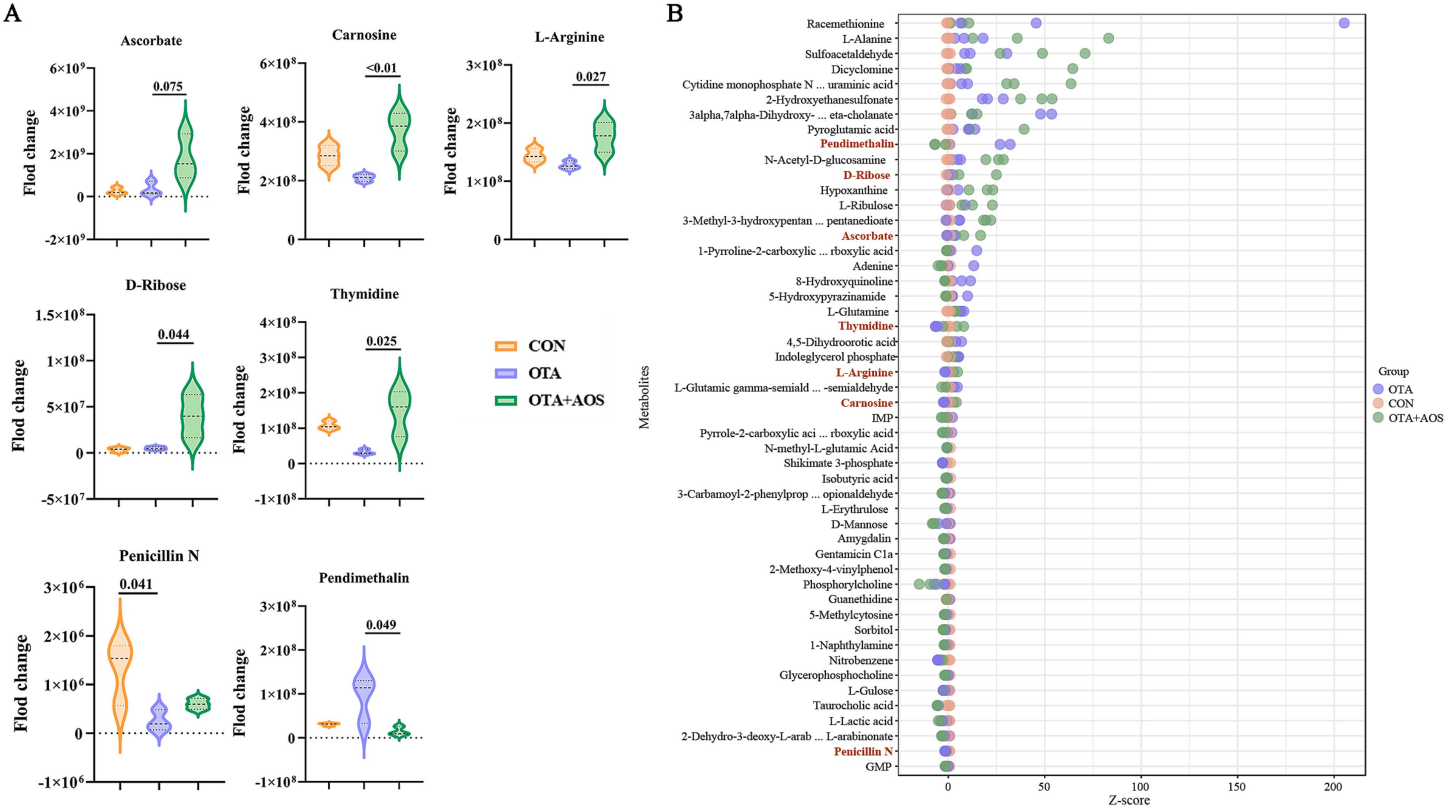
Effect of alginate oligosaccharides (AOS) on renal key differential metabolites in mice exposed to ochratoxin A (OTA). **(A)** Violin diagram of typical differential metabolites. **(B)** *Z*-score diagram of differential metabolites. CON, 1 mL/kg B.W. H_2_O + 1 mL/kg B.W. 0.1 M sodium bicarbonate; OTA, 1 mL/kg B.W. H_2_O + 1 mL/kg B.W. 0.1 M sodium bicarbonate + 250 μg/kg B.W. OTA; OTA + AOS, 1 mL/kg B.W. H_2_O + 1 mL/kg B.W. 0.1 M sodium bicarbonate + 250 μg/kg B.W. OTA + 400 mg/kg B.W. AOS.

**Figure 5:**
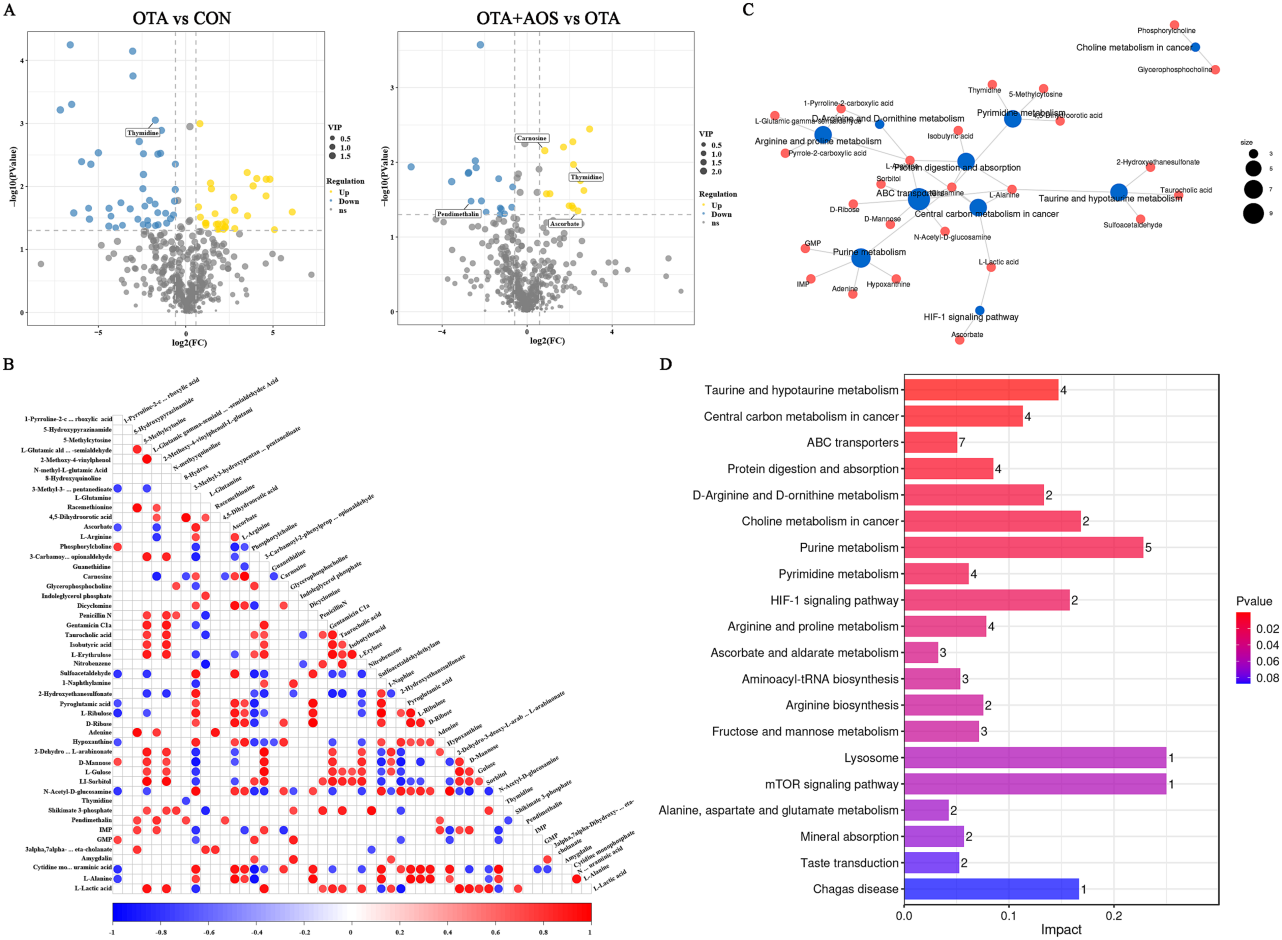
Effect of alginate oligosaccharides (AOS) on renal differential metabolites and pathway enrichment in mice exposed to ochratoxin A (OTA). **(A)** Volcanic diagram of OTA vs. CON and OTA + AOS vs. OTA differential metabolites. **(B)** Differential metabolite correlation thermogram. **(C)** KEGG enrichment analysis network diagram. **(D)** KEGG enrichment analysis bar chart. CON, 1 mL/kg B.W. H_2_O + 1 mL/kg B.W. 0.1 M sodium bicarbonate; OTA, 1 mL/kg B.W. H_2_O + 1 mL/kg B.W. 0.1 M sodium bicarbonate + 250 μg/kg B.W. OTA; OTA + AOS, 1 mL/kg B.W. H_2_O + 1 mL/kg B.W. 0.1 M sodium bicarbonate + 250 μg/kg B.W. OTA + 400 mg/kg B.W. AOS.

Partial least squares discriminant analysis (PLS-DA) model shows excellent discrimination between groups. Permutation test confirmed the validity of the model, and the intercept values *R*^2^ = 0.96 and *Q*^2^ = 0.29 in positive ion mode, *R*^2^ = 0.96 and *Q*^2^ = 0.22 in negative ion mode, indicating that there is no over-fitting (Figures 3A–F). As can be seen from Figures 3G,H, there are 79 different metabolites in OTA group and CON group, of which 33 are up-regulated and 46 are down-regulated. There were 36 differential metabolites in OTA + AOS group and OTA group, of which 15 were up-regulated and 21 were down-regulated. We screened the differential metabolites and found that compared with the CON group, the content of thymidine decreased in OTA group (VIP >1, *p* < 0.05). In addition, compared with OTA group, the contents of ascorbate, carnosine and thymidine increased after AOS supplementation (VIP >1, *p* < 0.05) (Figures 4A,B). In addition, among the differential metabolites, a characteristic peak of mass spectrometry presumed to be pendimethalin was significantly reduced after AOS supplementation. It is worth noting that the identification is a database matching result and needs to be verified later (VIP >1, *p* < 0.05) (Figures 5A,B). The KEGG pathway annotation of different metabolites revealed that they were mainly related to protein digestion and absorption, amino acid metabolism and mTOR signaling pathway (Figures 5C,D).

### 3.4 Effect of AOS on renal transcriptome in mice exposed to OTA

**Figure 6:**
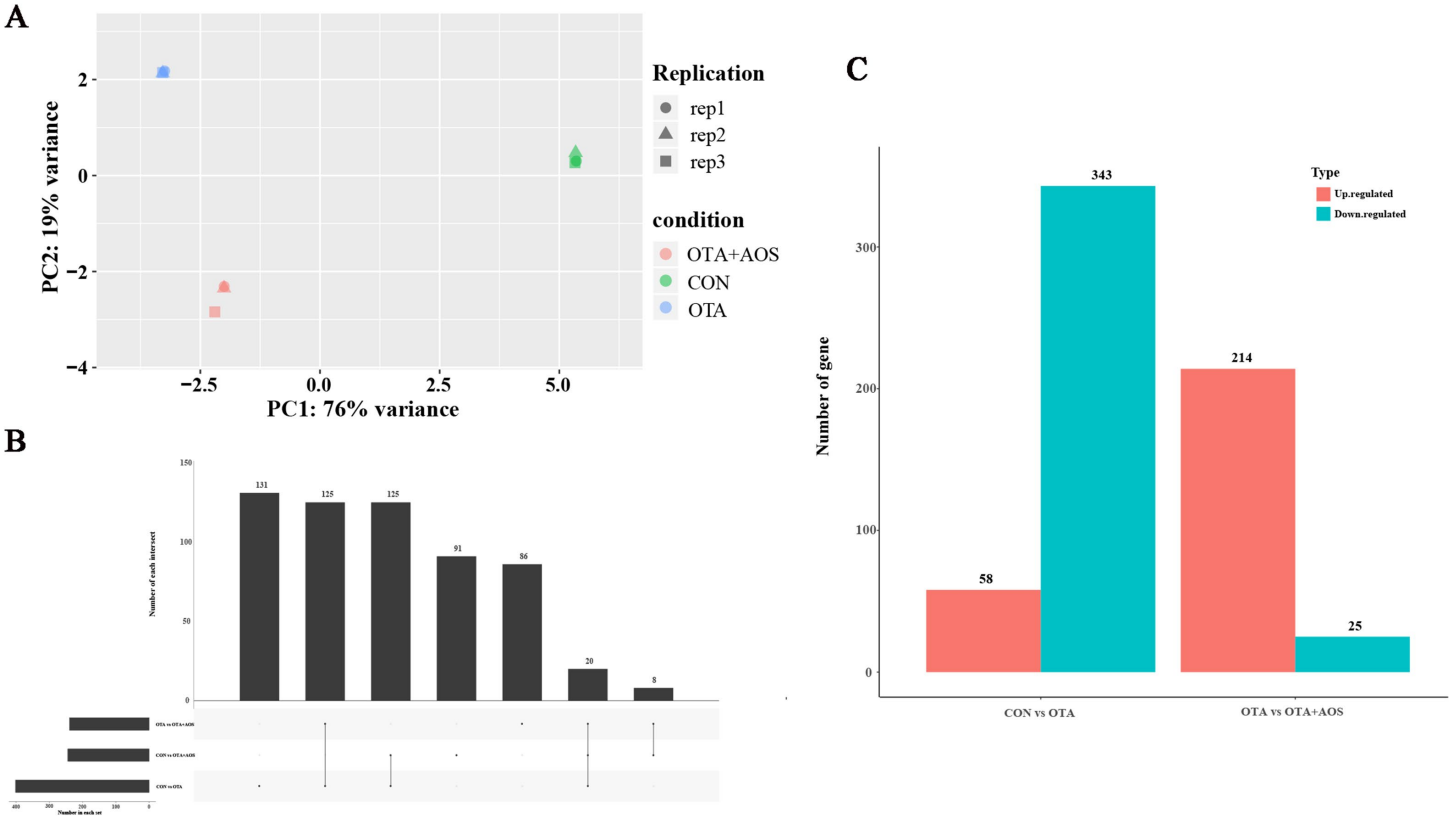
Effects of alginate oligosaccharides (AOS) on renal transcriptome of mice exposed to ochratoxin A (OTA). **(A)** Principal component analysis (PCA) diagram. **(B)** Upset map of differential genes between groups. **(C)** Differential gene Venn diagram. CON, 1 mL/kg B.W. H_2_O + 1 mL/kg B.W. 0.1 M sodium bicarbonate; OTA, 1 mL/kg B.W. H_2_O + 1 mL/kg B.W. 0.1 M sodium bicarbonate + 250 μg/kg B.W. OTA; OTA + AOS, 1 mL/kg B.W. H_2_O + 1 mL/kg B.W. 0.1 M sodium bicarbonate + 250 μg/kg B.W. OTA + 400 mg/kg B.W. AOS.

**Figure 7:**
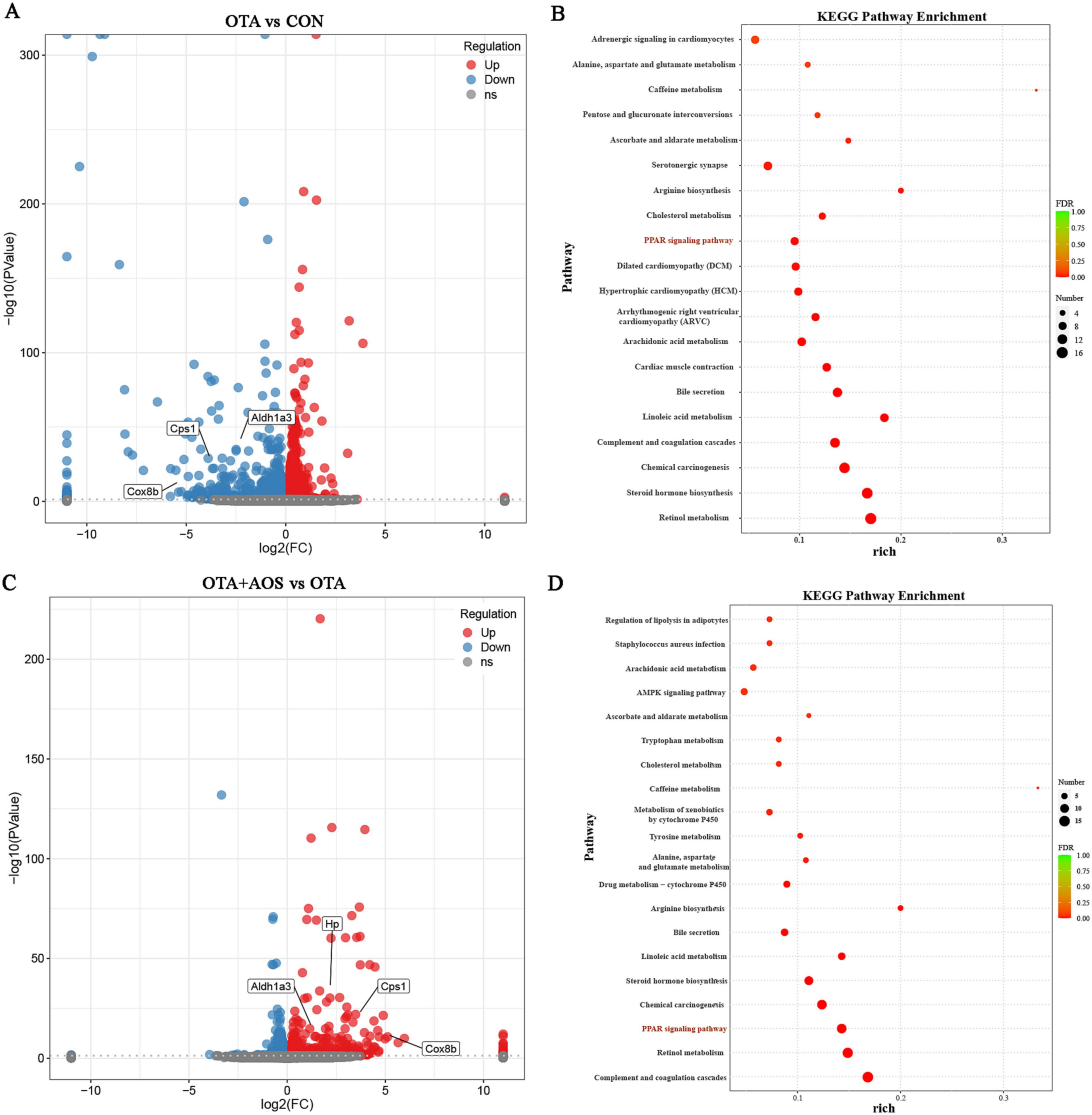
Effects of alginate oligosaccharides (AOS) on differential genes and pathways enrichment in the kidney of mice exposed to ochratoxin A (OTA). **(A)** Volcano map of OTA vs. CON groups. **(B)** KEGG enrichment bubble diagram of OTA vs. CON groups. **(C)** Volcano map of OTA + AOS vs. OTA groups. **(D)** KEGG enrichment bubble diagram of OTA + AOS vs. OTA groups. CON, 1 mL/kg B.W. H_2_O + 1 mL/kg B.W. 0.1 M sodium bicarbonate; OTA, 1 mL/kg B.W. H_2_O + 1 mL/kg B.W. 0.1 M sodium bicarbonate + 250 μg/kg B.W. OTA; OTA + AOS, 1 mL/kg B.W. H_2_O + 1 mL/kg B.W. 0.1 M sodium bicarbonate + 250 μg/kg B.W. OTA + 400 mg/kg B.W. AOS.

**Figure 8:**
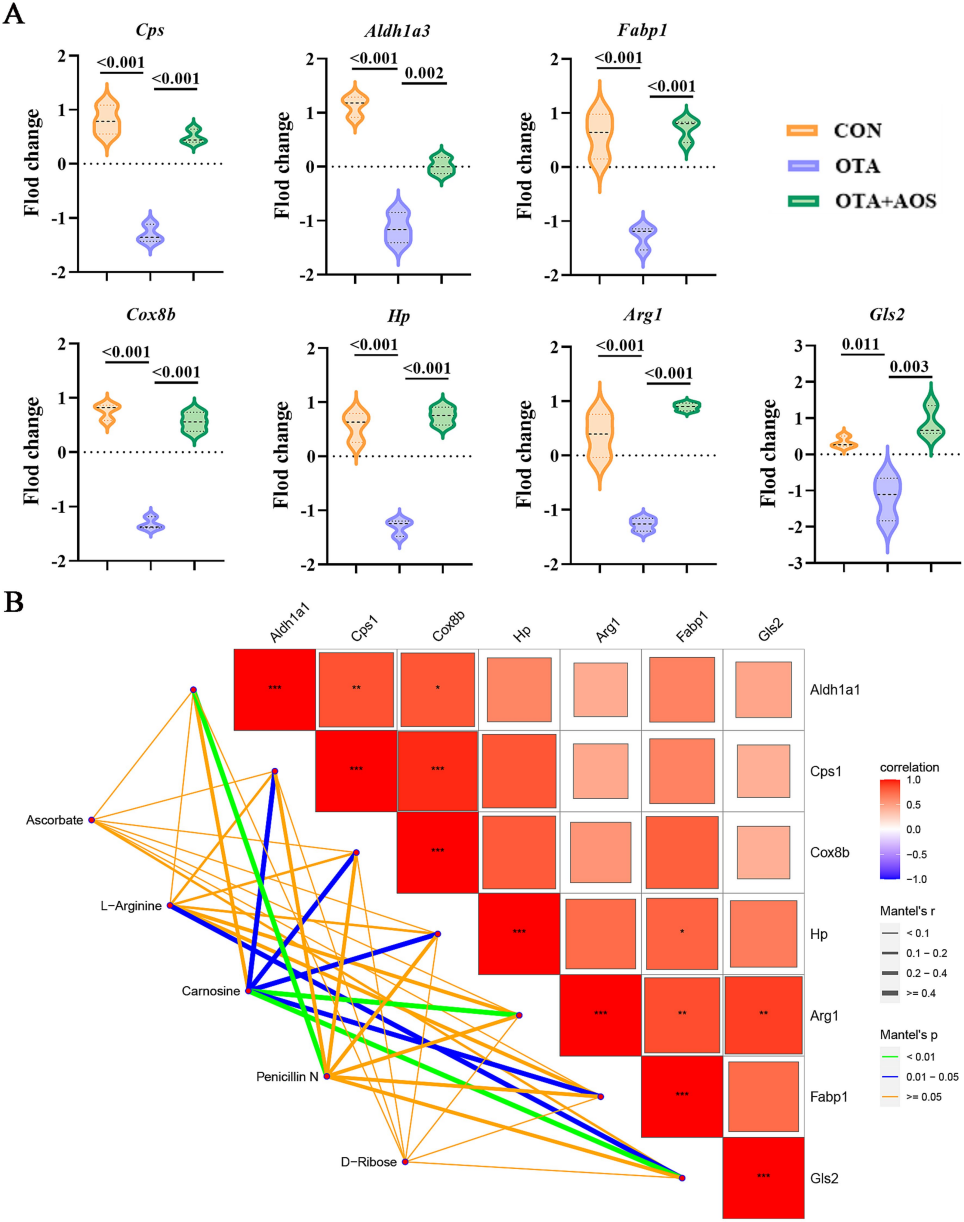
Effects of alginate oligosaccharides (AOS) on the correlation between differential genes and metabolites in the kidney of mice exposed to ochratoxin A (OTA). **(A)** Violin diagram of typical differential genes. **(B)** Mantel test correlation thermogram of differential metabolites and differential genes. CON, 1 mL/kg B.W. H_2_O + 1 mL/kg B.W. 0.1 M sodium bicarbonate; OTA, 1 mL/kg B.W. H_2_O + 1 mL/kg B.W. 0.1 M sodium bicarbonate + 250 μg/kg B.W. OTA; OTA + AOS, 1 mL/kg B.W. H_2_O + 1 mL/kg B.W. 0.1 M sodium bicarbonate + 250 μg/kg B.W. OTA + 400 mg/kg B.W. AOS.

To explore the effect of AOS on OTA-induced kidney in mice, the transcriptome of three kidney samples from CON, OTA and OTA + AOS groups was generated by Illumina high-throughput sequencing platform. principal component analysis (PCA) showed that there were obvious differences between these groups, demonstrating that the model establishment and drug intervention could influence the transcriptional profiles (Figure 6A). There were 401 differential genes in CON group and OTA group, of which 58 were up-regulated and 343 were down-regulated. There are 239 differential genes in OTA group and OTA + AOS group, of which 214 are up-regulated and 25 are down-regulated. There are 20 different genes among the three groups (Figures 6B,C). Volcano map showed that the expressions of aldehyde dehydrogenase 1 family member A3 (*Aldh1a3*), carbamoyl-phosphate synthase 1 (*Cps1*) and cytochrome c oxidase subunit 8B (*Cox8b*) in OTA group were lower than those in CON group (|log2 FC| >1, *p* < 0.05). In addition, compared with OTA group, the expressions of *Aldh1a3*, *Cps1*, *Cox8b* and haptoglobin (*Hp*) increased after AOS supplementation (|log2 FC| >1, *p* < 0.05) (Figures 7A,C, 8A). KEGG enrichment analysis showed that PPAR signaling pathway was significantly enriched among the three groups (Figures 7B,D).

### 3.5 Effect of AOS on the correlation between metabolomics and transcriptomes in mice exposed to OTA

Combined with the comprehensive analysis of metabolomics and transcriptomics results, oral administration of AOS on the basis of OTA can up-regulate the expression of *Cps*, *Aldh1a1*, *Cox8b*, *Gls2*, and increase the products such as L-arginine and carnosine, which is beneficial to its detoxification in renal cells (Mantel’s *p* < 0.05) (Figure 8B).

## 4 Discussion

OTA is a mycotoxin commonly found in feed, which can cause severe kidney injury in animals (26). Bzkurt et al. (27) found that the renal cortex of rats exposed to OTA was bleeding and hyperemia, accompanied by inflammatory cell infiltration. It was found that the exposure of OTA causes significant pathological damage to the kidney, especially the proximal tubule epithelial cells, which are sensitive targets, are widely swollen and vacuolated, and the brush border structure is blurred or disappeared (28). Our study found that OTA led to the blurring of balloon lumen, infiltration of renal cortical lymphocytes, and mild edema of renal tubular epithelial cells, which was similar to previous studies. This suggests that OTA can destroy the integrity of renal tubular structure and interfere with its reabsorption function, which is an important morphological basis for renal dysfunction. AOS intake improved the histological morphology of mouse kidney. As we all know, structure determines function. When the morphological structure of kidney tissue is damaged, its function will also be impaired (29). The present results show that AOS supplementation improves the histological damage of mouse kidney induced by OTA and reduce the infiltration of inflammatory cells. Similar to previous studies, it was found that AOS supplementation improves the glomerular and tubular functions of rats’ kidneys (30). At the subcellular level, the results of transmission electron microscope further revealed the specific damage of OTA to mitochondria. As the core site of cell energy metabolism, the structural disorder of mitochondria will inevitably lead to ATP synthesis disorder and aggravate the cell energy crisis. It is found that OTA leads to mitochondrial shrinkage, crista structure fracture, dissolution or even disappearance, brush edge damage, partial mitochondrial membrane integrity damage, showing vacuolar changes (31). Our study is similar to early observations, and it is found that OTA induces the fuzzy structure of mouse renal mitochondrial crista and the disappearance and fusion of mitochondrial bilayer membrane (32). In this study, AOS supplementation alleviates the damage of renal mitochondria ultrastructure induced by OTA. To sum up, OTA induces morphological changes in renal tissue, specifically damages the ultrastructure of mitochondria, destroys the homeostasis of cell energy metabolism and induces oxidative damage, which together constitute an important pathological basis of renal toxicity in mice. However, AOS supplementation can reverse this injury, which may be related to the natural structure and various biological functions of AOS.

Based on non-targeted metabolomics analysis, this research found that OTA exposure disturbs the renal metabolic homeostasis of experimental animals, and AOS supplementation effectively reverses the abnormal changes of key metabolites. Thymidine, as a key precursor of DNA synthesis and repair (33, 34), is significantly reduced in OTA group, suggesting that the proliferation and repair ability of kidney cells are impaired, which is consistent with the study of Gan et al. (35) in porcine kidney cells (PK15 cells)-OTA hinders DNA repair by inhibiting thymidine kinase activity. The recovery of AOS level after intervention may support renal tubular epithelial regeneration by promoting nucleic acid metabolism. The synchronous increase of ascorbate and carnosine after AOS supplementation has important physiological significance. Ascorbic acid is the main water-soluble antioxidant, and the increase of its content can explain the role of AOS in enhancing free radical scavenging ability, which is similar to previous study (36). The increase of carnosine may reduce the apparent genotoxicity by inhibiting histone modification (37), which provides a new perspective for the nephrotoxicity mechanism of OTA. There is another unexpected but worth discussing finding in this study: the level of a compound matching the mass spectrum characteristics of herbicide pendimethalin in OTA group increased. Although pendimethalin itself is not a known metabolite of OTA, the study shows that pendimethalin can induce mitochondrial damage and cell cycle arrest, which is highly coincident with the core toxic mechanism of OTA (38). Therefore, this phenomenon may suggest that OTA exposure unexpectedly disturbs a metabolic pathway that can produce functional molecules with similar toxicity, or leads to the accumulation of some endogenous metabolites with similar structure to pendimethalin. This provides a clue for a new hypothesis, but it needs to be strictly verified in future research through standard comparison and functional experiments. Our study found that pendimethalin accumulated in OTA group but decreased in AOS group, suggesting that OTA may interfere with the removal of exogenous poisons, while AOS may reduce the secondary toxicity burden by activating detoxification pathway. Kidney plays an important role in the synthesis, filtration, reabsorption and excretion of amino acids, which can retain useful metabolites and excrete potential harm and waste in amino acid metabolism (39). The orderly metabolism of amino acids in kidney is the central hub of body balance, and nephropathy will affect the level and metabolism of various amino acids (40). As a central hub for regulating autophagy and protein synthesis, the inhibition of mTOR signaling pathway by OTA will hinder the clearance and tissue repair of damaged organelles, and the regulation of this pathway by AOS may be a key mechanism for its nephroprotective effect (41, 42). Metabolomics analysis revealed the regulatory mechanism of AOS on the nephrotoxicity of OTA. To put it simply, AOS may reverse the metabolic imbalance induced by OTA through synergistic regulation of nucleic acid metabolism, antioxidant defense, poison clearance and mTOR-mediated cell steady-state reconstruction, which provides molecular evidence for explaining its renal protection mechanism.

In this study, the changes in kidney genes were investigated by transcriptomics. As a rate-limiting enzyme in urea cycle, the decrease of *Cps1* expression directly leads to ammonia detoxification disorder (43, 44), and the up-regulation of *Cps1* induced by AOS echoes the increase of L-arginine in metabolomics, which proves that AOS promotes ammonia clearance by activating urea cycle. COX8b is a mitochondrial complex, and its activation will enhance the activity and oxidative phosphorylation of mitochondria (45). After AOS supplementation, the recovery of *Cox8b* expression indicates that the function of mitochondrial respiratory chain is improved, which is confirmed by the repair of mitochondrial crista structure observed by electron microscope in the early stage. Up-regulation of *Aldh1a3* may affect tubular differentiation and regeneration by regulating retinoic acid signaling and enhancing renal antioxidant performance, and its mechanism of action is similar to the report of Yuan et al. (46), the nephroprotective effect is associated with the activation of ALDH family. PPAR signaling pathway, as a key metabolic control hub, not only acts as a core transcription factor for regulating fatty acid oxidation, but also plays a certain role in regulating mitochondrial energy homeostasis, amino acid metabolism and kidney antioxidant performance (47, 48). Multivariate association analysis further confirmed that AOS promotes the accumulation of protective metabolites such as L-arginine and carnosine by up-regulating *Cps1*, *Aldh1a3* and *Cox8b* simultaneously, which were enriched into PPAR signaling. Therefore, AOS can alleviate OTA nephrotoxicity through PPAR signaling hubs. However, it must be pointed out that the sample size of this study is small (*n* = 3/group), which is mainly limited by the cost of exploratory multivariate analysis. Although the omics data show strong biological consistency and coherent logic, this limitation means that the current conclusions need to be carefully interpreted and independently verified in large sample studies and the in-depth mechanism of action will be validated in the future researches.

## 5 Conclusion

In conclusion, oral administration of 400 mg/kg B.W. AOS improves the morphological damage of renal tissue and the changes of mitochondrial ultrastructure, up-regulates the expression levels of *Cps1*, *Aldh1a3* and *Cox8b*, and increases the accumulation of protective metabolites such as L-arginine and carnosine in mice exposed to OTA. Combined with the metabolomics and transcriptomics data, AOS alleviates OTA-induced nephrotoxicity in mice mainly through PPAR pathway. This study offers new insights into application of seaweed-derived saccharides to alleviate OTA-induced kidney damage, which is beneficial for the development of functional detoxifying agents from marine sources. Besides, this study provides preliminary omics clues, and the in-depth mechanism of action will be validated in the future researches.

## Data Availability

The original contributions presented in the study are included in the article/Supplementary material, further inquiries can be directed to the corresponding author.

## Footnotes

1
http://ccb.jhu.edu/software/hisat2/index.shtml
