# Supplementary material for: Multi-omics analysis reveals that alginate oligosaccharides mitigate ochratoxin A-induced renal impairment in mice and is relevant to the regulation of PPAR signaling
Source: Front Vet Sci. 2026 Jan 12;12:1702799. doi: 10.3389/fvets.2025.1702799 (PMC12832446; doi:10.3389/fvets.2025.1702799)
Supplement: Supplementary file 1 [file Table_1.docx]

| **Table S1** **Comparative analysis of key parameters among Control, OTA, and OTA+AOS groups.** | | | | | |
| --- | --- | --- | --- | --- | --- |
| Parameter | Control | OTA | OTA+AOS | Statistic | P value |
| Mitochondrial number  (cells, ×5.0K) | 16.33±2.19 | 11.00±0.58 | 10.33±0.88 | F=5.51 | 0.044 |
| Mitochondrial average area  (μm2, ×5.0K) | 5.67±0.12 | 2.00±0.02 | 7.33±0.16 | H=5.96 | 0.025 |
| Ascorbate  (Relative Abundance) | 2.55*10^8^±9.23*10^7^ | 3.40*10^8^±1.90*10^8^ | 1.78*10^8^±6.06*10^8^ | F=5.34 | 0.047 |
| Carnosine  (Relative Abundance) | 2.86*10^8^±1.97*10^7^ | 2.10*10^8^±6.96*10^6^ | 3.72*10^8^±3.75*10^7^ | F=10.67 | 0.011 |
| L-Arginine  (Relative Abundance) | 1.44*10^8^±6.77*10^6^ | 1.28*10^8^±3.84*10^6^ | 1.76*10^8^±1.47*10^7^ | F=6.61 | 0.030 |
| D-Ribose  (Relative Abundance) | 3.58*10^6^±1.38*10^6^ | 5.08*10^6^±9.66*10^5^ | 4.00*10^7^±1.34*10^7^ | F=6.98 | 0.027 |
| Thymidine  (Relative Abundance) | 1.08*10^8^±6.93*10^6^ | 3.23*10^7^±4.10*10^6^ | 1.46*10^8^±3.73*10^7^ | F=6.92 | 0.028 |
| Penicillin N  (Relative Abundance) | 1.30*10^6^±3.73*10^5^ | 2.49*10^5^±1.23*10^5^ | 6.02*10^5^±6.43*10^4^ | F=5.43 | 0.045 |
| Pendimethalin  (Relative Abundance) | 3005*10^7^±1.79*10^6^ | 9.22*10^7^±3.01*10^7^ | 1.47*10^7^±5.71*10^6^ | F=5.33 | 0.047 |
| *Cps*  (Normalized Counts) | 0.806±0.15 | -1.302±0.10 | 0.495±0.07 | F=100.70 | <0.001 |
| *Aldh1a3*  (Normalized Counts) | 1.128±0.11 | -1.141±0.16 | 0.013±0.09 | F=83.32 | <0.001 |
| *Cox8b*  (Normalized Counts) | 0.757±0.09 | -1.317±0.07 | 0.560±0.10 | F=177.80 | <0.001 |
| *Hp*  (Normalized Counts) | 0.561±0.16 | -1.308±0.12 | 0.747±0.12 | F=93.25 | <0.001 |
| *Fabp1*  (Normalized Counts) | 0.590±0.24 | -1.287±0.12 | 0.697±0.12 | F=42.63 | <0.001 |
| *Arg1*  (Normalized Counts) | 0.372±0.23 | -1.268±0.07 | 0.896±0.04 | F=64.76 | <0.001 |
| *Gls2*  (Normalized Counts) | 0.335±0.09 | -1.199±0.34 | 0.865±0.24 | F=18.72 | 0.003 |
